# Benign Symptomatic Squamous Papilloma of the Uvula: A Case Report and Review of the Literature

**DOI:** 10.7759/cureus.33602

**Published:** 2023-01-10

**Authors:** Ethar A Albasry, Zahra M Alyousef, Mohammed A Alazizi

**Affiliations:** 1 General Practice, Dammam Medical Complex, Dammam, SAU; 2 General Practice, Qatif Central Hospital, Dammam, SAU; 3 Otolaryngology - Head and Neck Surgery, Almana Group of Hospitals, Dammam, SAU

**Keywords:** benign tumors, globus dysphagia, uvula, human papillomavirus (hpv), squamous papilloma

## Abstract

Squamous papillomas are benign lesions that are associated with human papillomavirus infection. Oral squamous papilloma of the uvula is uncommon and rarely causes symptoms. In this case report, we present a rare case of symptomatic squamous papilloma of the uvula where the patient complained of mass-related symptoms such as dysphagia and choking sensation. The lesion was surgically excised using electrocautery with excellent outcomes. In addition, we reviewed literature related to the lesion.

## Introduction

Oral squamous papillomas (OSPs) are asymptomatic neoplasms of the oral cavity, growing mostly in the soft palate [1]. Although these neoplasms are benign, their appearance is worrisome when they mimic other malignant oral lesions [2]. In rare cases, some patients present with disturbing symptoms such as dysphagia, odynophagia, and globus sensation; in serious conditions, patients might present with obstructive symptoms, especially if the lesion has a substantial overgrowth [3]. The definitive diagnosis depends on clinical and histopathological examinations [1]. Treatment modalities differ; however, treatment mainly relies on the surgical removal of the lesion [3]. In this case report, we present a rare case of symptomatic squamous papilloma of the uvula growing up to 2 cm. The lesion was excised using electrocautery with excellent outcomes.

## Case presentation

A 24-year-old female presented with a complaint of dysphagia to solids and odynophagia for 2 months. These symptoms started progressively and were associated with continuous throat clearing, globus sensation, and on-and-off choking sensation, especially during sleep. Upon examination, a long pedicled lesion was noticed extending from the uvula. Flexible nasopharyngoscopy revealed an elongated uvula touching the posterior part of the tongue. Post-nasal drip and acid reflux were noticed in the pharyngeal cavity. No ulceration or discoloration was seen on the lesion.

**Figure 1:**
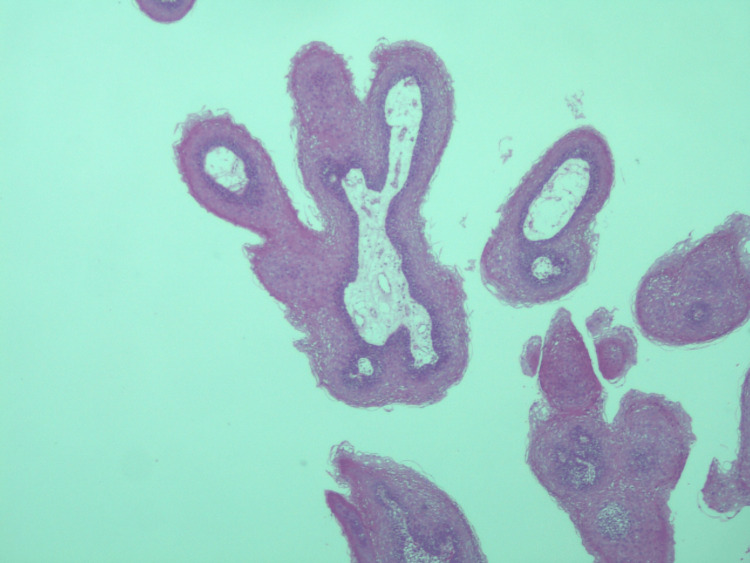
Low-power image of the biopsy shows exophytic lesions with finger-like projections, showing cores lined by benign stratified squamous epithelium

**Figure 2:**
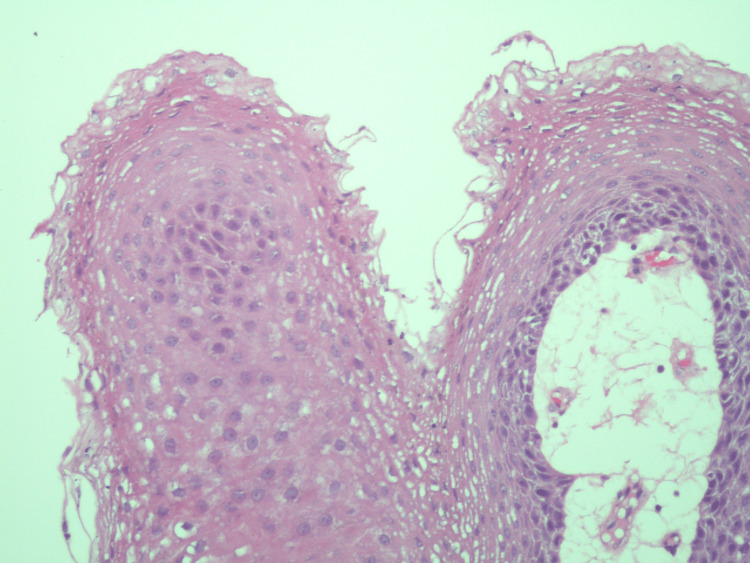
High-power image shows squamous cells and fibrovascular cores consistent with benign features

The patient underwent surgery under general anesthesia, in which the lesion was excised using electrocautery. A macroscopic histopathology examination revealed a soft tissue measuring 2 cm x 0.5 cm x 0.3 cm. Microscopic findings showed a polyp lined by benign stratified squamous epithelium (Figure 1). The stroma showed collagen and stellate cells - results consistent with squamous cell papilloma (Figures 1, 2). The patient’s postoperative course remains unremarkable with complete resolution of symptoms, even though she had been discharged on oral proton pump inhibitors for acid reflux, oral antihistamines, and steroid inhalers.

## Discussion

OSPs are benign, asymptomatic soft tissue neoplasms of the oral cavity. Their macroscopic appearance is characterized by pedunculated, usually, solitary exophytic extensions with a cauliflower-like surface [2]. It is reported that OSPs are associated with human papillomavirus (HPV) especially HPV-6, HPV-11, and HPV-16 and predominantly occur in the second to fourth decades [3].

Symptomatic squamous papillomas are rare. The location and size of the lesion play a role in the emergence of symptoms [1,4]. Five similar case reports of symptomatic uvular papillomas have been found in the literature (Table 1). Less than 25% of oral squamous papillomas are greater than 1 cm [5]. However, only one of the five cases was reported to be greater than 1.5 cm, all of which had similar mass-related symptoms. In this study, the patient had a relatively large mass of about 2 cm.

**Table 1:** Review of related case reports

Reference	Gender	Age	Symptoms	Size	Location	Modality of treatment
[3]	Male	21	Sore throat, odynophagia, irritation on the posterior part of the tongue	1 cm x 1 cm	Uvula	Electrocautery
[4]	Male	43	Airway obstructive symptoms	0.8 cm x 0.9 cm x 0.5 cm	Uvula	Electrocautery
[5]	Male	10	Dysphagia and choking sensation	3 cm x 2 cm	Uvula	Electrocautery
[6]	Female	18	Dysphagia, choking sensation, globus sensation, and frequent throat clearing	0.5 cm x 0.5 cm x 0.5 cm	Uvula	Electrocautery
[7]	Female	22	Dysphagia, choking sensation, globus sensation, frequent throat clearing, dry cough, excess mucus, and heartburn	1.5 cm	Uvula	Electrocautery

In a case series involving 464 cases of oral papillomas, 34% of the cases were in the palatal complex (hard palate, soft palate, tongue, and uvula). Only 4% of the cases were located in the uvula [1].

Another retrospective study was conducted to analyze 207 biopsies of oral papillomas performed from 1996 to 2012. Most of the lesions occurred in the soft palate (23%). The second most common site was the posterior border of the tongue (20.9%). The lesions were more predominant in males than females (ratio = 1.26:1) [2].

Along with the clinical evaluation, the definitive diagnosis of squamous papillomas depends on biopsy and histopathological examination [6]. Features include fibrovascular cores lined with benign stratified squamous epithelium [1-3]. The treatment mainly depends on surgical interventions. Different modalities include electrocautery, laser ablation, cold-steel excision, cryosurgery, or intra-lesional injections of interferon [1,3,7].

## Conclusions

Symptomatic squamous papillomas are influenced by the location. For instance, uvular papilloma can be associated with symptoms due to its distinct site of occurrence. Although it is rare, uvular squamous papilloma is reported to be associated with mass-related symptoms such as dysphagia and choking sensation. Although lesions greater than 1 cm are few, even small-sized uvular papillomas appear to cause symptoms. The treatment of choice predominantly relies on electrocautery.
